# Higher-Order Hamiltonian for Circuits with (*α*,*β*) Elements

**DOI:** 10.3390/e22040412

**Published:** 2020-04-05

**Authors:** Zdeněk Biolek, Dalibor Biolek, Viera Biolková, Zdeněk Kolka

**Affiliations:** 1 Department of Microelectronics, Brno University of Technology, 616 00 Brno, Czech Republic; zdenek.biolek@gmail.com (Z.B.); dalibor.biolek@unob.cz (D.B.); 2 Department of Electrical Engineering, University of Defence, 662 10 Brno, Czech Republic; 3 Department of Radio Electronics, Brno University of Technology, 616 00 Brno, Czech Republic; kolka@feec.vutbr.cz

**Keywords:** higher-order element, constitutive relation, Hamiltonian, Lagrangian, Chua’s table, memristor, Euler-Lagrange equation

## Abstract

The paper studies the construction of the Hamiltonian for circuits built from the (*α*,*β*) elements of Chua’s periodic table. It starts from the Lagrange function, whose existence is limited to Σ-circuits, i.e., circuits built exclusively from elements located on a common Σ-diagonal of the table. We show that the Hamiltonian can also be constructed via the generalized Tellegen’s theorem. According to the ideas of predictive modeling, the resulting Hamiltonian is made up exclusively of the constitutive relations of the elements in the circuit. Within the frame of Ostrogradsky’s formalism, the simulation scheme of Σ-circuits is designed and examined with the example of a nonlinear Pais–Uhlenbeck oscillator.

## 1. Introduction

**Figure 1:**
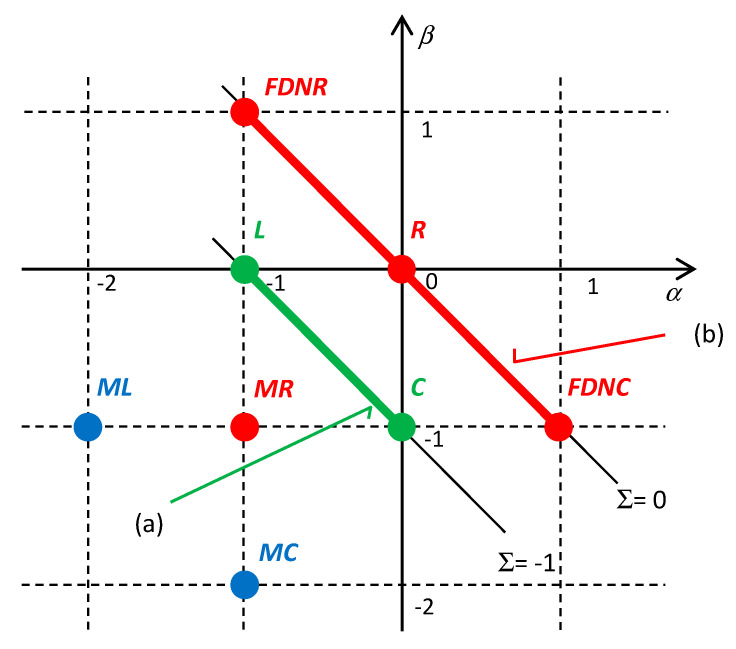
Visualization of several (*α*,*β*) elements in Chua’s table. *R*, *L*, *C* = Resistor, Inductor, and Capacitor; *MR*, *ML*, *MC* = Memristor, Meminductor, and Memcapacitor; *FDNR*, *FDNC* = Frequency Dependent Negative Resistor and Frequency Dependent Negative Conductor. (a) Lagrange’s formalism of classical mechanics applies to circuits composed exclusively of *L* and *C* elements; the energy, conserved in the circuit, corresponds to the diagonal Σ = −1; (b) an example of the application of a 2^nd^-order Lagrangian to the description of the Pais–Uhlenbeck oscillator [15] consisting of dissipative *R*, *FDNR*, *FDNC* elements [16]; the power preserved in the circuit corresponds to the diagonal Σ = 0.

The dynamics of complex nonlinear systems attracts the interest of researchers in various branches of science [1,2,3,4]. One of the approaches used for studying system dynamics, predictive modeling, considers the system an interconnection of one-port devices, the *α*,*β* elements, also known as Higher Order Elements (HOEs) [5]. Each HOE guarantees that its terminal quantities are coupled in all circumstances via a constitutive relation. In electrical engineering, the terminal quantities are the generalized voltage *v*^(^*^α^*^)^ and current *i*^(^*^β^*^)^, where the integers *α* and *β* denote the orders of the derivative/integral with respect to time (for positive/negative integers). Well-known (*α*,*β*) elements include resistors (0,0), capacitors (0,−1), and inductors (−1,0), and also the promising memristors (−1,−1), memcapacitors (−1,−2), and meminductors (−2,−1), frequency-dependent resistors (*FDNR*, *FDNC*), and others. HOEs are usually represented as points with integer (*α*,*β*) coordinates in Chua’s periodic table (see Figure 1). The constitutive relation of the element is frequently written in one of the two following forms:

(1) $$v^{\left(\alpha \right)}=f\left(i^{\left(\beta \right)}\right),\;\mathrm{or}\;i^{\left(\beta \right)}=g\left(v^{\left(\alpha \right)}\right)$$

where the function *f*() or *g*() models the current or voltage representation of the element.

The use of HOEs considerably increases the possibilities of analyzing and synthesizing circuits with complex dynamic behavior. Thanks to predictive modeling, many phenomena can be better understood. For example, the hypothetical element called a memristor [6] helped to explain the peculiar hysteresis behavior observed when measuring various nanomaterials. When Hewlett-Packard reported the first TiO_2_-based working memristive device in 2008 [7], they reminded the researchers of the original work [6] that explains this behavior as a characteristic feature of the memristor—a fingerprint. Ongoing research has demonstrated that a pinched hysteresis loop is a regular phenomenon, which takes effect for an arbitrary (*α*,*β*) element in the space of the time derivatives of its terminal quantities [8].

The interest in systems with high-order dynamics has led to an extension of Lagrange’s and Hamilton’s formalisms to systems with *α*,*β* elements. This is because the original Lagrange’s formalism starts from the fundamental schematic of classical mechanics, i.e., from the point of view of predictive modeling based on the *R* (0,0), *L* (−1,0), and *C* (0,−1) elements. Moreover, the system of equations of motion can be generated from one scalar function, the Lagrangian, but only for conservative systems. Recent works [9,10,11] have extended Lagrange’s formalism to circuits containing memristors, memcapacitors, and meminductors. The following work [12] defines the potential functions for general (*α*,*β*) elements from which Lagrangians and dissipative functions are drawn, and rules for generating the equations of motions are established. The potential functions of a general (*α*,*β*) element are defined for current or voltage representation as integrals:

(2) $$S_{\alpha ,\beta }=\int v^{\left(\alpha \right)}di^{\left(\beta \right)}\;\mathrm{or}\;{\hat{S}}_{\alpha ,\beta }=\int i^{\left(\beta \right)}dv^{\left(\alpha \right)},$$

which represent a natural generalization of the potential and kinetic energy and the dissipative function of classical mechanics, or the energy of capacitors, inductors, and the content of resistors in classical electrical engineering. Two different representations of potential functions correspond to the concept of functions and co-functions introduced by Millar [13] and Cherry [14] in 1951.

The classical Lagrangian is of the first order, so it is a function of generalized coordinates and velocities (first-order time derivatives of the coordinates); the resulting equations of motion are of the second order. Higher-order Lagrangians were introduced by Ostrogradsky in [17] as follows:

(3) $$L=L\left(x,x^{\left(1\right)},\ldots ,x^{\left(m\right)},t\right)$$

The Lagrangian is a function of the 1x*n* vector of the generalized coordinates ***x*** = [*x*_1_ .. *x_n_*]^T^ and their derivatives up to the integer-order *m*. The system represented by Lagrangian (3) is governed by *n* equations of motion:

(4) $$\frac{\partial L}{\partial x}-\frac{d}{dt}\left(\frac{\partial L}{\partial x^{\left(1\right)}}\right)+\frac{d^{2}}{dt^{2}}\left(\frac{\partial L}{\partial x^{\left(2\right)}}\right)-\ldots +{\left(-1\right)}^{m}\frac{d^{m}}{dt^{m}}\left(\frac{\partial L}{\partial x^{\left(m\right)}}\right)=0.$$

Equation (4) is a direct consequence of the fact that the system trajectory is extremal to the action

(5) $$A=\int_{t_{1}}^{t_{2}}L\left(x,x^{\left(1\right)},\ldots ,x^{\left(m\right)},t\right)dt,$$

which is the essence of Hamilton’s variational principle [18]. The higher-order Lagrangian (3) was used for the first time to describe circuits built from HOEs in [16]. This work demonstrated that the Lagrangian, which has the ability to generate equations of motion, can only be drawn for the Σ-circuits that contain only elements from the common Σ-diagonal of Chua’s table, where the sum of the indices *α* and *β* is preserved; thus, Σ = *α* + *β*. The Lagrangian then has the form

(6) $$L=\sum_{\varepsilon_{i}}{\left(-1\right)}^{i}S_{i}\;\mathrm{or}\;\hat{L}=\sum_{\varepsilon_{i}}{\left(-1\right)}^{i}{\hat{S}}_{i}.$$

**Figure 2:**
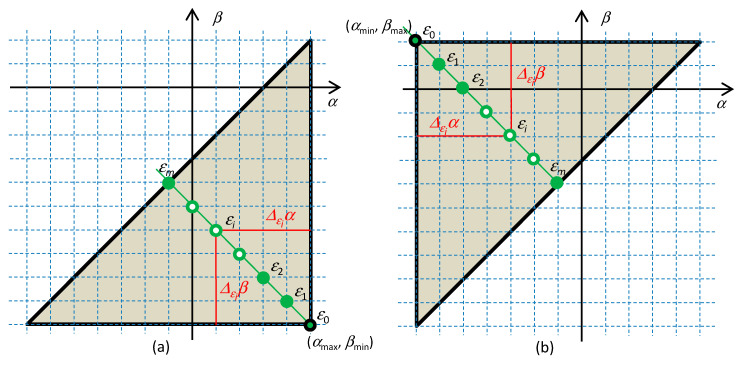
This circuit consists of elements of *ε*_0_ to *ε_m_* types, which are located on the common Σ-diagonal. Characteristic quarter-circles are constructed for the (a) current and (b) voltage representation of the circuit. Since the elements are located on the Σ-diagonal, their distances from the hidden element are the same in both the *α* and *β* directions; thus, Δ*_εi_α* = Δ*_εi_β* = *i*.

The summation is done through all *ε_i_* elements of the given Σ-diagonal, where *i* is the position of the element on the diagonal (see Figure 2). The signs in front of the state functions in the sums of (6) are governed by the type (even or odd) of the positions of the elements on the diagonal [16]. The difference between kinetic and potential energy, transferring between the inertial and the accumulating elements, which are the common form of the Lagrangian used in classical mechanics, are merely a special case of (6). The physical dimension of the Lagrangian is given by the number Σ of the diagonal: [Volt⋅Amper⋅sec^−^^Σ^]. This is the energy [Volt⋅Amper⋅sec] in *LC* and the power [Volt⋅Amper] in resistive circuits. An example of the Lagrangian of the Pais–Uhlenbeck oscillator [15], consisting of three resistive elements of *R*, *FDNR*, and *FDNC* types, is given in [16].

The transition from the higher-order Lagrangian (3) to the higher-order Hamiltonian is given by the generalized Legendre transformation published by Ostrogradsky in [17]:

(7) H(x,x(1),‥,x(2m−1))=pT1⋅x(1)+…+pTm⋅x(m)−L(x,x(1),…,x(m))

where ^1^***p*** to *^m^**p*** are vectors of the generalized momenta

(8) pj=∑k=jm(−1)k−j(∂L∂x(k))(k−j).

Consider the notation

(9) q1=x, q2=x(1),…,qm=x(m−1).

Assume that the Lagrangian is regular, i.e.,

(10) $$\det\left(\frac{\partial^{2}L}{\partial x_{i}^{\left(m\right)}\partial x_{j}^{\left(m\right)}}\right)\neq 0,$$

for *i*,*j* = 1,...,*n* [19]. In Equation (8), for *j* = *m* reads as

(11) pm=∂L∂x(m).

Then, the unambiguous relation,

(12) x(m)=χ(q1,…,qm,pm),

can be derived from (11), which enables a transition to new coordinates ^1^***p***,.., *^m^**p*** and ^1^***q***,..., *^m^**q*** and allows us to draw the canonic equations,

(13) q˙j=∂H∂pj, p˙j=−∂H∂qj.

The Hamiltonian *H* is then a function of the new variables *^i^**q***, *^i^**p***, *i* = 1, ... , *m*:

(14) H(q1,…,qm,p1,…,pm)=pT1⋅q2+…+pTm−1⋅qm+pTm⋅χ(q1,…,qm,pm)−L(q1,…,qm,pm)

where

(15) L(q1,…,qm,pm)=L(q1,…,qm,χ(q1,…,qm,pm)).

The procedure of drawing an alternative Hamiltonian for cases when condition (10) of the regularity of the Lagrangian is not fulfilled is described, for example, in [19]. Irrespective of whether the Lagrangian is or is not regular, the Hamiltonian can be used within Lagrange’s formalism, i.e., in the sense of generalized energy (7).

This formalism, specific to higher-order Hamiltonians of type (14), is denoted Ostrogradsky’s formalism [19]. None of the hitherto published works deal with assembling the Hamiltonian of systems consisting of elements from Chua’s table. The benefits of using the Hamiltonian have been proven: the system dynamics is given by a set of canonic first-order differential equations, which are the standard starting point for solving complex tasks associated with terms such as the Lyapunov exponents, zero divergence, conservative phase volumes [20], etc. Hamiltonians are an ideal tool when searching for symmetries and associated conservative quantities [21]. Today, they are well defined also for systems that have hitherto been considered non-conservative [22].

The objective of this work is therefore to find the Hamiltonian of a Σ-system consisting of arbitrary HOEs within the frame of both Lagrange’s formalism (as generalized energy) and Ostrogradsky’s formalism. The conventional procedure for introducing Ostrogradsky’s formalism into physical theory involves searching for the form of the Lagrangian and generalized momenta, and subsequently obtaining from them a specific form of the Hamiltonian ((7) or (14)). The procedure used in this work will be physically more objective, starting from the generalized Tellegen’s theorem. In addition to its clarity, the reason for selecting this approach is pragmatic: Tellegen’s theorem is extremely general and applicable to circuits of arbitrary topologies with any elements (linear, nonlinear, time-invariant, and time-varying), as well as with arbitrary elements from Chua’s table. Moreover, this theorem can also be formulated via the terminal quantities of individual elements. It is useful for circuits containing HOEs, since the sole fundamental characteristic of the element—the constitutive relation—is a link only between these terminal quantities. Then, the resulting Hamiltonian will naturally be comprised of the constitutive relations of all HOEs in the circuit.

This paper has the following structure. Section 2 introduces the generalized form of Tellegen’s theorem applicable to circuits with HOEs and thus to circuits with the elements defined via the constitutive relations (1). Section 3 is devoted to drawing a specific form of the Hamiltonian for Σ-circuits in the frame of Lagrange’s formalism and also of Ostrogradsky’s formalism. Section 4 describes a modeling technique inspired by Ostrogradsky’s formalism. In the last section, these new pieces of knowledge are applied to a specific topology of the Pais–Uhlenbeck oscillator consisting of HOEs.

## 2. Tellegen‘s Theorem for Circuits with Higher-Order Elements

Consider a circuit comprised of arbitrary one-port elements *ε* with port voltages *v_ε_* and currents *i_ε_*. Then, the generalized Tellegen’s theorem [23] holds for such a circuit:

(16) $$\sum_{\varepsilon }v_{\varepsilon }^{\left(\alpha \right)}i_{\varepsilon }^{\left(\beta \right)}={\left(v_{\varepsilon }^{\left(\alpha \right)}\right)}^{T}\cdot i_{\varepsilon }^{\left(\beta \right)}=0$$

where *α* and *β* are arbitrary integers, $v_{\varepsilon }^{\left(\alpha \right)}$ and $i_{\varepsilon }^{\left(\beta \right)}$ are 1x*b* vectors of generalized voltages and currents of the element, and *b* is the number of elements in the circuit. The classical form of the theorem, representing the case *α* = *β* = 0, means that the sum of instantaneous powers delivered to all elements in the circuit is zero. The generalized theorem (16) replaces the instantaneous power [VA] by a quantity [VA]*^α^* ^+^ *^β^*. This is because the integration and differentiation with respect to time belong to Kirchhoff’s operators [19], which do not affect the validity of Kirchhoff’s laws and the theorems derived from them. Note that the source of the voltage *v*^(^*^α^*^)^ or current *i*^(^*^β^*^)^ can be substituted by an (*α*,*β*) element with a constant constitutive relation (1) of a *f*() or *g*() type, so theorem (16) also holds for circuits with voltage and current sources.

Consider a circuit comprised of general HOEs. The element *ε_h_* = (*α*_max_,*β*_min_) is the hidden element of the current representation of the circuit [24]. Let us introduce new variables *u* = *v*^(^*^α^*^max)^ and *x* = *i*^(^*^β^*^min)^. Then, Equation (16) can be rewritten with the aid of Equation (1) in the form

(17) $$\sum_{\varepsilon }f_{\varepsilon }^{\left(\alpha +\alpha_{\max}-\alpha_{\varepsilon }\right)}x_{\varepsilon }^{\left(\beta +\beta_{\varepsilon }-\beta_{\min}\right)}=0$$

where *f_ε_* and *x_ε_* are the constitutive relation (1) and the *x* variable of the element *ε*. Since Equation (16) must hold for arbitrary integers *α* and *β*, it also holds for *α* = 0 and *β* = 1 + *β*_min_ − *β_ε_*. Substituting these values in formula (17), which is identically equal to zero, and integrating it with respect to time, the resulting quantity must be constant in all circumstances:

(18) $$H=\int \sum_{\varepsilon }f_{\varepsilon }^{\left(\Delta_{\varepsilon }\alpha \right)}{\dot{x}}_{\varepsilon }dt=const$$

where Δ*_ε_α* = *α*_max_ − *α_ε_* is the distance of the *ε* element from the hidden element in the *α* direction (see Figure 2a).

A similar procedure leads to the dual form of Equation (18) for voltage representation of the circuit:

(19) $$H^{*}=\int \sum_{\varepsilon }g_{\varepsilon }^{\left(\Delta_{\varepsilon }\beta \right)}{\dot{u}}_{\varepsilon }dt=const^{*}$$

where Δ*_ε_β* = *β_ε_* − *β*_min_ is the distance of the *ε* element from the hidden element *ε_h_* = (*α*_min_,*β*_max_) in the *β* direction (see Figure 2b).

It will be shown in the following section that the quantity (18) or (19) is, for the case of Σ-circuits, a Hamiltonian of type (7) and that it becomes a Hamiltonian of type (14) after transitioning to coordinates that correspond to Ostrogradsky’s formalism.

## 3. From Tellegen‘s Theorem to the Hamiltonian

Let all the elements of the circuit be located on a common Σ-diagonal according to Figure 2. Denote such a circuit as the Σ-circuit. All types of elements will be specified as *ε*_0_ to *ε_m_*, where *m* is the distance of the *ε_m_* element from the hidden element in the *α* or *β* direction (since the element is located on the Σ-diagonal, these distances are the same, thus Δ*_ε_α* = Δ*_ε_β* =Δ*_ε_*). The method of indexing depends on the position of the hidden element, and this position depends on the choice between the current, *f*(), and the voltage, *g*(), representation (1) of the element. The relation *ε*∈*ε_i_*, *i*=0,...,*m* will thus specify that the given *ε* element is only of the *ε_i_* type.

Let us focus on the proof that the formula (18) for the current representation of elements (see Figure 2a) is a Hamiltonian. The proof for the formula (19), which corresponds to the voltage representation, is analogous, so, for the sake of brevity, it will not be given below.

The sum (18) for *ε*∈*ε*_0_ is equal to

(20) $$\sum_{\varepsilon \in \varepsilon_{0}}\int f_{\varepsilon }^{\left(0\right)}{\dot{x}}_{\varepsilon }dt=\sum_{\varepsilon \in \varepsilon_{0}}\int f_{\varepsilon }^{\left(0\right)}dx_{\varepsilon }=\sum_{\varepsilon \in \varepsilon_{0}}S_{\varepsilon }.$$

For *ε*∈*ε_m_*, *m* > 0, repeated integration by parts leads to the following result:

(21) $$\sum_{\varepsilon \in \varepsilon_{m}}\int f_{\varepsilon }^{\left(m\right)}{\dot{x}}_{\varepsilon }dt=\sum_{\varepsilon \in \varepsilon_{m}}\left(\sum_{j=0}^{m-1}{\left(-1\right)}^{j}f_{\varepsilon }^{\left(m-1-j\right)}x_{\varepsilon }^{\left(j+1\right)}\right)+\sum_{\varepsilon \in \varepsilon_{m}}{\left(-1\right)}^{m}S_{\varepsilon }.$$

Summing for all the circuit elements and rearranging the terms yield the conservative quantity (18) in a compact form:

(22) $$H=\sum_{j=1}^{m}{\left(-1\right)}^{j-1}\left(\sum_{i=j}^{m}\sum_{\varepsilon \in \varepsilon_{i}}x_{\varepsilon }^{\left(j\right)}f_{\varepsilon }^{\left(i-j\right)}\right)+\sum_{i=0}^{m}\sum_{\varepsilon \in \varepsilon_{i}}{\left(-1\right)}^{i}S_{\varepsilon }.$$

The generalized currents *x_ε_* can be represented as linear combinations of generalized loop currents *x*. Utilizing incidence matrices, the addend of the inner summation of the first term in (22) will assume the form

(23) ∑k=1naεkxk(j)fε(i−j)=∑k=1nxk(j)fε(i−j)k

where *^k^f_ε_* means either ±*f_ε_* or 0 depending on whether the element *ε* is or is not a part of the *k*-th loop, and, possibly, depending on what its orientation is with regard to this loop. Substituting (23) into (22), expanding the outer series according to index *j*, and subsequently rearranging the summation yield

(24) H=∑k=1n(xk(1)∑i=1m∑ε∈εi(fε(i−1)k)︸pk1+xk(2)∑i=2m∑ε∈εi(− fε(i−2)k)︸pk2+…+xk(m)∑ε∈εm((−1)m−1fε(0)k)︸pkm)−∑i=0m∑ε∈εi(−1)iSε︸L

Since it holds that

(25) ∂L∂xk=(−1)k+1fεk,

comparing (24) and (7) reveals that the function *ℋ* from Equation (24) is the generalized energy (7) of the current representation of a circuit with HOEs, and the generalized momenta are

(26) pkj=(−1)j−1∑i=jm∑ε∈εi(fε(i−j)k).

The one-dimensional case for *n* = 1 signifies that all the elements are in series, with the common generalized current *x*. The series connection of the elements of the same type can, therefore, be regarded as one element of the same type, with the constitutive relation given as the sum of constitutive relations of the individual elements. Equation (26) can, therefore, be rewritten in the simplified form

(27) pj=(−1)j−1∑i=jmfi(i−j)(x(i))

where *f_i_*() denotes the constitutive relation of an element of the *ε_i_* type. The generalized momenta are then given as summations of the time derivatives of the constitutive relations

(28) p1=f1(0)+f2(1)+‥+fm(m−1)p1=f1(0)+f2(1)+‥+fm(m−1)⋮pm=(−1)m−1fm(0).

This one-dimensional case clearly illustrates the transition from the formulation of the Hamiltonian (7) to the formulation (14) by changing the coordinates (*x*^(0)^,..., *x*^(*m*)^) of Lagrange’s formalism to new coordinates (^1^*p*,...,^*m*^*p*, ^1^*q*,...,^*m*^*q*) of Ostrogradsky’s formalism. The key relation *χ*() from (12) can be obtained via a simple inversion of the constitutive relation of the element of the *ε_m_* type:

(29) pm=(−1)m−1fm(x(m))⇒x(m)=gm((−1)m−1pm)

where *g_m_*() is the constitutive relation of the voltage representation of the element of the *ε_m_* type.

## 4. Modeling of the Σ-Circuits

Ostrogradsky’s formalism leads to a system of canonic first-order equations (13). This fact facilitates the modeling process. It follows from (28) that the momenta of a one-dimensional system are

(30) p˙1=−f0(q1)p˙2=−p1+f1(q2)p˙3=p2+f2(q3)⋮p˙m=(−1)m−1 pm−1+fm−1(qm).

Similarly, the generalized coordinates are

(31) q˙1=q2q˙2=q3⋮q˙m−1=qmq˙m=gm((−1)m pm).

**Figure 3:**
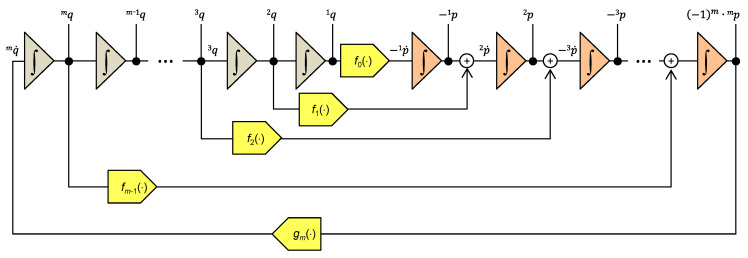
Model of the Σ-system according to Ostrogradsky’s formalism. The color-codes represent the *m* integrators for computing the *^i^q* coordinates and the *m* integrators for computing the *^i^p* momenta. The function blocks model the constitutive relations of individual elements. The *g_m_*() block is for modeling the inverse function of *f_m_*() according to (29), which enables the transition from Lagrange’s to Ostrogradsky’s formalism.

The systems of Equations (30) and (31) lead to the elegant programming diagram in Figure 3, consisting of 2x*m* integrators for computing *m* generalized momenta and *m* generalized coordinates, as well as *m*+1 function blocks for modeling the constitutive relations of the individual elements.

## 5. Application: Non-Linear Pais–Uhlenbeck Oscillator

The usefulness of the Hamiltonian for the analysis of HOE circuits can be demonstrated by the example of the Pais–Uhlenbeck (PU) oscillator [15]. The PU oscillator is frequently employed to test new physical theories. It generates a signal with two harmonic components whose amplitudes and initial phases are given by the initial conditions. Both frequencies are coupled via the formula [25]

(32) $$\omega^{\pm }=\omega \sqrt{\frac{1\mp \sqrt{1-4\epsilon }}{2\epsilon }}$$

where ϵ is a real number, 0 ≤ ϵ < ¼. For ϵ → 0, the PU oscillator changes to a classical sinusoidal oscillator with an oscillation frequency of *ω*.

The PU oscillator is governed by the differential equation

(33) $$\frac{\epsilon }{\omega^{2}}\ddddot{x}+\ddot{x}+\omega^{2}x=0.$$

In general, the signal *x* generated by the oscillator is non-periodical. Periodicity is achieved in special cases when the ratio of both frequencies is a rational number. The corresponding values of the constant ϵ are as follows:

(34) $$\epsilon =\frac{1}{4}\left(1-{\left(\frac{1-k^{2}}{1+k^{2}}\right)}^{2}\right),\;k=\frac{\omega^{+}}{\omega^{-}}=\frac{r}{s}<1$$

where *r*, *s* are integers. Only in these cases will the state-space trajectories be closed curves.

**Figure 4:**
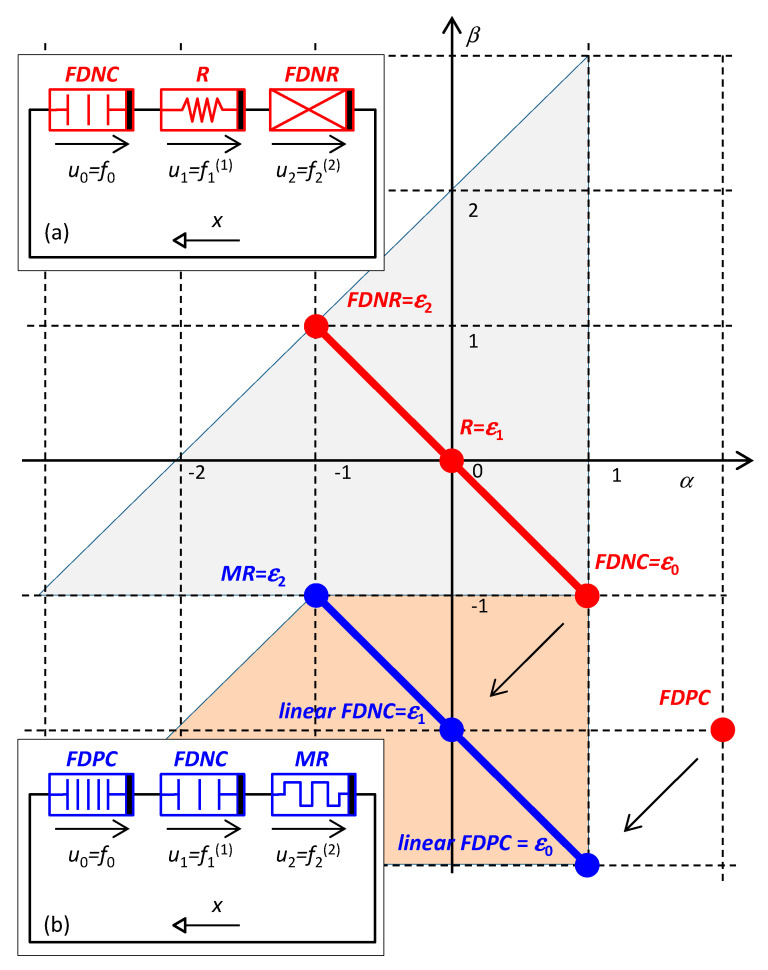
Schematics and element layout in Chua’s table for two different implementations of the Pais–Uhlenbeck (PU) oscillator (a) / (b): The *FDNC* / *linear FDPC* element [26] with the coordinates (*α*_max_,*β*_min_) = (1,−1) / (1,−3) is the hidden element of the oscillator. The hidden element determines the physical nature of the generalized quantities: The generalized current *x* is the charge *q*=*i*^(−1)^ / double integral o charge *i*^(−3)^, the generalized voltages *u* are derivatives of the element voltages with respect to time *v*^(1)^. The arrows between the pairs of elements in Chua’s table denote that, for linear constitutive relations, both elements play the same role in the circuit and are thus interchangeable. Insets: implementations of the PU oscillator via three Higher Order Elements (HOEs) in series.

It is shown in [16] that the PU oscillator, modeled by Equation (33), can be implemented as a series or parallel Σ-circuit consisting of an arbitrary triad of HOEs, which are immediate neighbors on an arbitrary Σ-diagonal. One possible combination is shown in the inset in Figure 4a, namely, the series connection of the *FDNC*, *R*, and *FDNR* elements with the constitutive relations *f*_0_(), *f*_1_(), and *f*_2_(). In general, these relations can be nonlinear. The indices 0 to 2 correspond to the distance of the element from the hidden element in the *α* or *β* direction. The hidden element is the *FDNC*. The generalized current *x* is governed by the differential equation

(35) $${\ddot{f}}_{2}\left(\ddot{x}\right)+{\dot{f}}_{1}\left(\dot{x}\right)+f_{0}\left(x\right)=0$$

If all three elements have linear constitutive relations,

(36) $$f_{0}\left(x\right)=\omega^{2}x,\;f_{1}\left(\dot{x}\right)=\dot{x},\;f_{2}\left(\ddot{x}\right)=\frac{\epsilon }{\omega^{2}}\ddot{x},$$

then the generalized current will be modeled via the linear differential Equation (33).

The subsequent analysis is made for linear *FDNC* and *R* and nonlinear *FDNR* elements with the following constitutive relations:

(37) $$f_{0}\left(x\right)=k_{0}x,\;f_{1}\left(\dot{x}\right)=k_{1}\dot{x},\;f_{2}\left(\ddot{x}\right)=k_{2}\arctan\ddot{x}.$$

The Lagrangian of the circuit is

(38) $$L\left(x,\dot{x},\ddot{x}\right)=-\frac{1}{2}k_{0}x^{2}+\frac{1}{2}k_{1}{\dot{x}}^{2}-\frac{1}{2}k_{2}\left(2\ddot{x}\arctan\ddot{x}-\ln\left(1+{\ddot{x}}^{2}\right)\right).$$

The equation of motion, generated by the Lagrangian according to (4), takes the form

(39) $$k_{0}x+k_{1}\ddot{x}+k_{2}\frac{\ddddot{x}\left(1+\ddot{x}\right)-{\dddot{x}}^{2}}{{\left(1+\ddot{x}\right)}^{2}}=0.$$

According to (28), the generalized momenta are

(40) p1=k1x˙+k2x⃛1+x¨2p2=−k2arctanx¨.

For Lagrange’s formalism, the Hamiltonian (7) will be in the form

(41) $$H\left(x,\dot{x},\ddot{x},\dddot{x}\right)=\frac{1}{2}k_{0}x^{2}+\frac{1}{2}k_{1}{\dot{x}}^{2}+k_{2}\frac{\dot{x}\dddot{x}}{1+{\ddot{x}}^{2}}-\frac{1}{2}k_{2}\ln\left(1+{\ddot{x}}^{2}\right).$$

In conformity with (9), we introduce the new variables

(42) q1=x, q2=x˙.

Utilizing Equation (40) to formulate the second derivative of *x* via the momentum ^2^*p* and slightly rearranging the equation will lead to the Hamiltonian

(43) H(q1,q2,p1,p2)=p1⋅q2+12k0⋅q21−12k1⋅q22+k2ln(cos(p2k2)).

**Figure 5:**
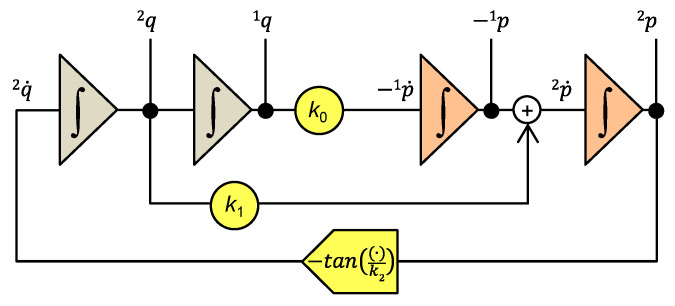
Modeling of the nonlinear PU oscillator within Ostrogradsky’s formalism.

The programming diagram for the nonlinear Pais–Uhlenbeck oscillator is shown in Figure 5.

**Figure 6:**
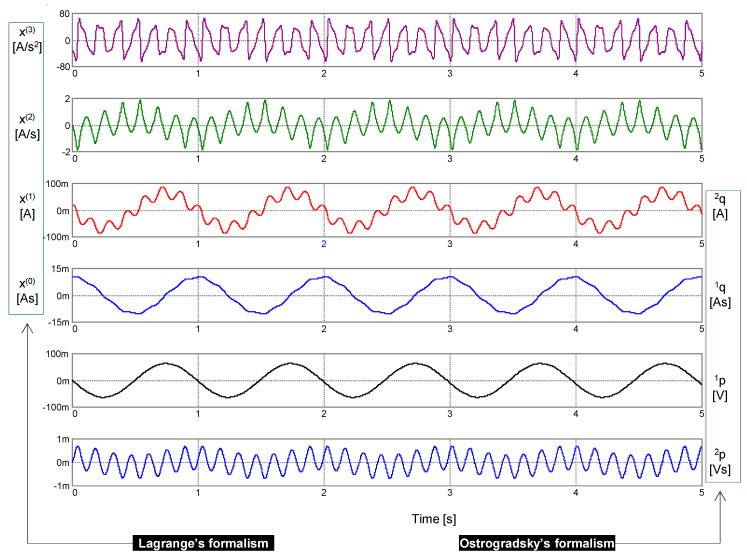
Waveforms of left-side legends: signal *x* generated via the PU oscillator together with its time derivatives of the first to third order *x*^(1)^ to *x*^(3)^. Waveforms with the right-side legends: the generalized coordinates ^1^*q* and ^2^*q* and the generalized momenta ^1^*p* and ^2^*p*. Parameters of the constitutive relations (37): *k*_0_ = (2π)^2^, *k*_1_ = 1, *k*_2_ = (2π)^−2^ ϵ, ϵ = 25.4. Initial conditions: Lagrange’s formalism—*x*(0) = 10 mAs, *x*^(1)^(0) = 20 mA, *x*^(2)^(0) = 0, *x*^(3)^(0) = −31.088 As^−2^; Ostrogradsky’s formalism—^1^*q*(0) = 10 mAs, ^2^*q*(0) = 20 mA, ^1^*p*(0) = 31 μV, ^2^*p*(0) = 0.

The results of a computer simulation are shown in Figure 6. Independent variables as arguments of the generalized energy ℋ (*x*, *x*^(1)^, *x*^(2)^, *x*^(3)^) (41) and the Hamiltonian *H* (^1^*q*, ^2^*q*, ^1^*p*, ^2^*p*) (43) are graphically differentiated.

The parameters of the oscillator and initial conditions are selected such that the oscillation performs at the fundamental frequencies of 1 Hz and 7 Hz. The phase trajectories are, therefore, closed curves. For the sake of completeness, the value of ϵ does not correspond to the frequency ratio 1:7, as provided by formula (34) because (34) holds for the linear PU oscillator, while in the experiment, a nonlinear *FDNR* was used.

**Figure 7:**
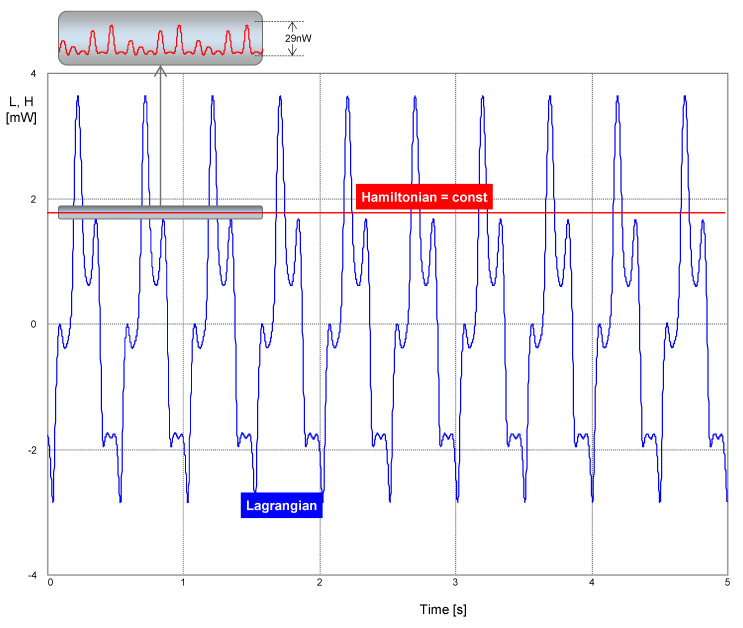
Lagrangian and Hamiltonian vs. time. During the movement, the value of the Hamiltonian was fixed at 1.775 mW. Inset: The negligible error in computing the Hamiltonian was achieved via a proper choice of the simulation parameters. A step ceiling of 500 μs was used for the adaptive time step.

Figure 7 displays the waveforms of the Lagrangian and the Hamiltonian. While the Lagrangian is time-varying, the Hamiltonian, as a conservative quantity, holds its initial value, which is given by the initial conditions (see the legend in Figure 6). According to Equation (41) or (43), this value is 1.775 mW, which conforms with the simulation. Due to time discretization, the numerical integration causes a seeming loss of circuit conservatism [27]. The inset in Figure 7 shows negligible deflections of the computed Hamiltonian from its theoretical constant value.

**Figure 8:**
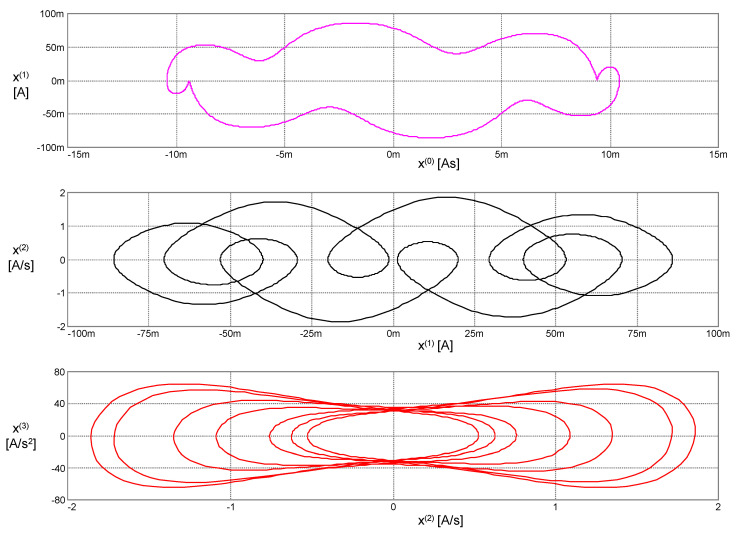
Phase trajectories of the PU oscillator in the coordinates according to Lagrange’s formalism.

The phase trajectories in Figure 8 represent the contours of the generalized energy (41): The conditionℋ(*x*, *x*^(1)^, *x*^(2)^, *x*^(3)^) = const holds during the motion along these trajectories.

**Figure 9:**
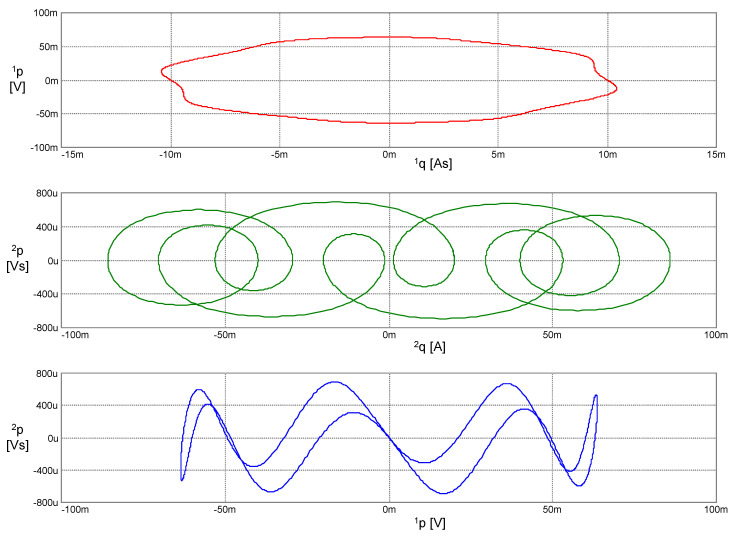
Phase trajectories of the PU oscillator in coordinates according to Ostrogradsky’s formalism.

The Hamiltonian (43) remains constant during motion. The phase trajectories from Figure 9, therefore, represent the contours of the Hamiltonian *H*: The condition *H*(^1^*q*, ^2^*q*, ^1^*p*, ^2^*p*) = const holds during the motion along these trajectories.

Another possible implementation of the PU oscillator is shown in Figure 4b. The oscillator is built from the memristor *MR* and the linear (0,−2) and (1,−3) elements. Utilizing the MOVE transformation [24] in Chua’s table and the duality principle, the two last mentioned elements can be replaced by their linear versions of *FDNC* and *FDPC* (Frequency Dependent Positive Conductance). The coordinates of the hidden element (*FDPC*) indicate that the generalized current *x* is a threefold time integral of the current, or *i*^(−3)^. Equation (35) now expresses the KV^(1)^L, where *f*_0_, *f*_1_, and *f*_2_ are the constitutive relations of the *FDPC*, *FDNR,* and *MR* elements.

Consider linear *FDPC* and *FDNR* elements in series with the HP (Hewlett-Packard) memristor [7], with the nonlinear dopant drift being modeled via the Joglekar window function with the parameter *p* = 1 [28]. The constitutive relations will be in the form [29]

(44) $$f_{0}\left(x\right)=k_{0}x,\;f_{1}\left(\dot{x}\right)=k_{1}\dot{x},\;f_{2}\left(\ddot{x}\right)=R_{off}\ddot{x}-\frac{\Delta R}{4k}\ln\left(\frac{A\exp\left(4k\ddot{x}\right)+1}{B}\right)$$

where *k*_0_, *k*_1_ are positive constants, Δ*R* = *R_off_*−*R_on_* is the difference between the maximum and the minimum memristance, *A* = (*R_off_*−*R_ini_*)/(*R_ini_*−*R_on_*), *B* = Δ*R/*(*R_ini_*−*R_on_*), *R_ini_* is the initial memristance, and *k* is a technological parameter [28]. The Lagrangian of the oscillator is generated after the integration of the constitutive relations and their appropriate summation:

(45) $$L\left(x,\dot{x},\ddot{x}\right)=-\frac{1}{2}k_{0}x^{2}+\frac{1}{2}k_{1}{\dot{x}}^{2}+\frac{1}{2}R_{off}{\ddot{x}}^{2}+\frac{\Delta R}{4k}\ddot{x}\ln B+\frac{\Delta R}{{\left(4k\right)}^{2}}Li_{2}\left(-A\exp\left(4k\ddot{x}\right)\right)$$

where *Li*_2_() denotes the dilogarithm [30], a special form of a polylogarithm, or Jonquière’s function.

The generalized momenta (28) are now

(46) p1=k1x˙+(Ron+ΔRAexp(4kx¨)+1)x⃛.p2=−Roffx¨+ΔR4klnAexp(4kx¨)+1B

For Lagrange’s formalism, the Hamiltonian (7) is

(47) $$H\left(x,\dot{x},\ddot{x},\dddot{x}\right)=\frac{1}{2}k_{0}x^{2}+\frac{1}{2}k_{1}{\dot{x}}^{2}-\frac{1}{2}R_{off}{\ddot{x}}^{2}+\frac{\Delta R}{4k}\ddot{x}\ln\left(A\exp\left(4k\ddot{x}\right)+1\right)+\;+\frac{\Delta R}{{\left(4k\right)}^{2}}Li_{2}\left(-A\exp\left(4k\ddot{x}\right)\right)+\left(R_{on}+\frac{\Delta R}{A\exp\left(4k\ddot{x}\right)+1}\right)\dot{x}\dddot{x}.$$

Applying Ostrogradsky’s formalism, the Hamiltonian acquires the form

(48) H(q1,q2,p1,p2)=p1⋅q2+12k0⋅q12−12k1⋅q22++ΔR(4k)2Li2(−Aexp(4k g2(p2)))+g2(p2)(p2+ΔR4klnB+12Roffg2(p2))

where *g*_2_() is, according to (31), the inverse of the constitutive relation *f*_2_() of the memristor.

The modeling diagram is the same as in Figure 5, but the functional block in the feedback will now correspond to the function *g*_2_(). The inverse of *f*_2_(), according to (44), cannot be derived in terms of standard functions. However, the inversion can be done numerically during the simulation run in SPICE. It follows from (44) that

(49) $$\ddot{x}=h(f_{2},\ddot{x})=\frac{f_{2}}{R_{off}}+\frac{\Delta R}{4kR_{off}}\ln\left(\frac{A\exp\left(4k\ddot{x}\right)+1}{B}\right).$$

Based on the known value *f*_2_, the simulation program computes $\ddot{x}$ from Equation (49) via iterations according to the SPICE code in Table 1.

**Figure 10:**
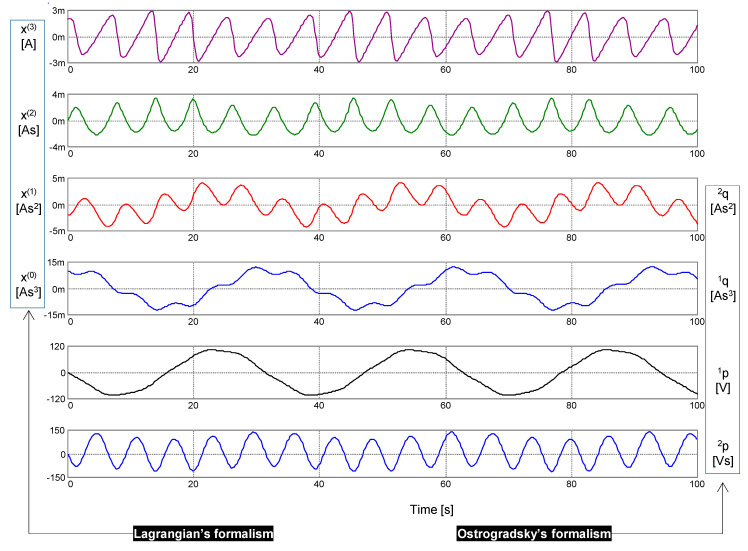
Waveforms of the basic quantities of a PU oscillator compounded of a memristor and linear *FDNC* and *FDPC* elements. The notation is the same as in Figure 6. Parameters of constitutive relations (44): *k*_0_ = (2π⋅7)^2^, *k*_1_ = 5e4, *k* = 111.11, *R_off_* = 100kΩ, Δ*R* = 99.99kΩ, *A* = 1, *B* = 2. Initial conditions: Lagrange’s formalism—*x*(0) = 10 mAs^3^, *x*^(1)^(0) = −2 mAs^2^, *x*^(2)^(0) = 0, *x*^(3)^(0) = 2 mA; Ostrogradsky’s formalism—^1^*q*(0) = 10 mAs^3^, ^2^*q*(0) = −2 mAs^2^, ^1^*p*(0) = −19.3 μV, ^2^*p*(0) = −100 μVs.

The simulation results are summarized in Figure 10.

The parameters of the elements and simulation options are again set such that the generated waveforms are periodical. The phase trajectories are, therefore, closed curves.

**Figure 11:**
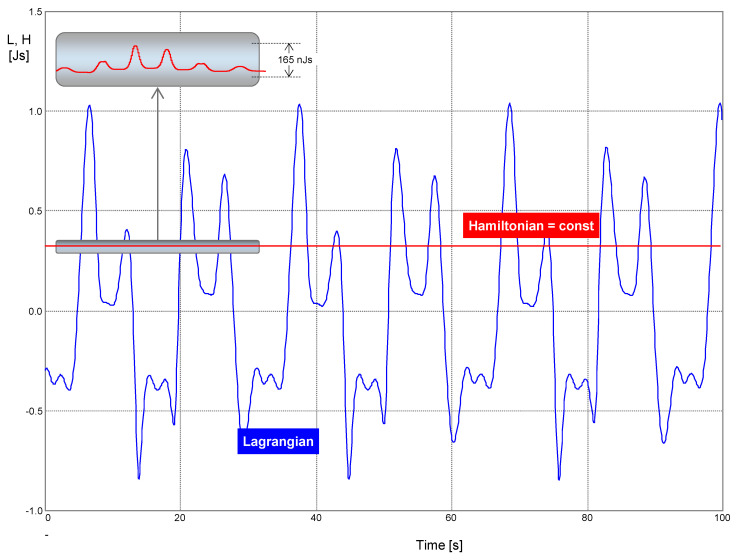
Lagrangian and Hamiltonian vs. time.

Figure 11 displays the waveforms of the Lagrangian and the Hamiltonian. The Hamiltonian is fixed at 296.3 mJs with small parasitic variation, which is minimized via the proper choice of simulation options. The step ceiling is 1 ms.

**Figure 12:**
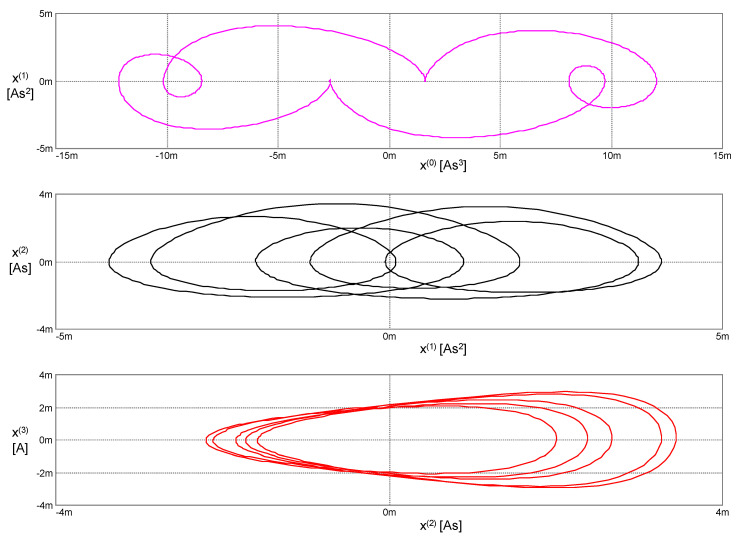
Phase trajectories of the PU oscillator with the HP memristor in the coordinates according to Lagrange’s formalism.

**Figure 13:**
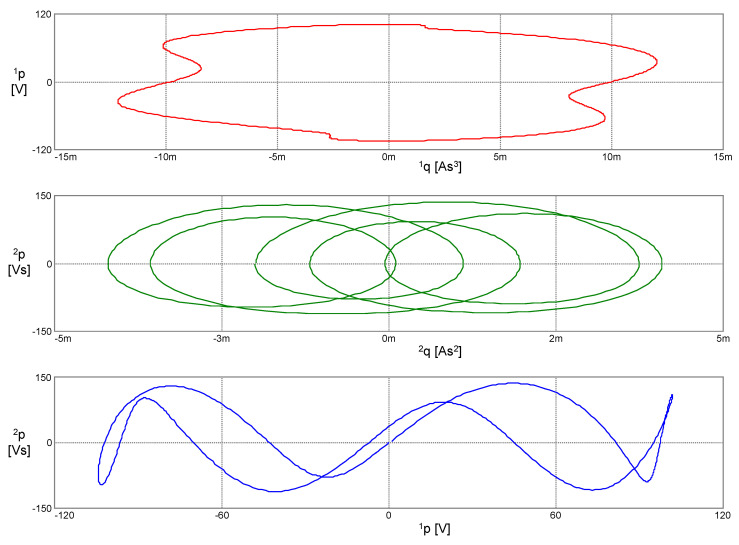
Phase trajectories of the PU oscillator with the HP memristor in the coordinates according to Ostrogradsky’s formalism.

The phase trajectories in Figure 12 and Figure 13 represent the contours of the generalized energy (47) and Hamiltonian (48). They can be discussed the same way as the trajectories in Figure 8 and Figure 9.

## 6. Discussion

Hamilton’s variational principle, i.e., that the trajectory is the extremal of Lagrange’s function, holds for the Σ-circuits [16]. This work offers new knowledge, namely that a Hamiltonian can be constructed for Σ-circuits and that this Hamiltonian preserves its fixed value during motion. Its physical dimension is given by the number Σ of the diagonal, and its unit is [VAs^—^^Σ^]. The Hamiltonian of a circuit consisting of generally nonlinear inductors and capacitors, therefore, represents the energy because both the inductor and capacitor are located on the diagonal with the number Σ = −1. The physical character of the Hamiltonian does not change even if other types of elements from this diagonal appear in the circuit.

The work in [9] was the beginning of this type of research. This study introduced the state functions of memristors, memcapacitors, and meminductors and constructed a Lagrangian from those functions. The research in [11,12,16], which appeared later, introduced the conditions for validating Hamilton’s variational principle, building up Hamilton’s formalism, and generalizing all these fundamental pieces of knowledge for general higher-order elements. It was demonstrated in [16] that the higher-order Lagrangian is “native” for general HOEs. Our work offers another insight: For this Lagrangian we construct the corresponding Hamiltonian, which is “native” for circuits with HOEs.

In order to derive benefits from Hamilton’s formalism in circuits with HOEs, we should use Ostrogradsky’s formalism because only the higher-order Hamiltonian is native for such circuits. One of its advantages is the ability to construct comfortable analyses of the preserved quantities when finding periodical steady states or the conditions of the occurrence of self-oscillation, as demonstrated by the example of the PU oscillator. The synthesis of this circuit via nonlinear HOEs leads to a nonlinear oscillator, which has not been hitherto described in the literature. The Hamiltonian of the PU oscillator consisting of the *FDNR*, *R*, and *FDNC* elements, according to Figure 4a, represents the power because these elements are located on the diagonal with Σ = 0. The Hamiltonian of the PU oscillator consisting of *MR*, *linear FDNC*, and *linear FDPC* elements, according to Figure 4b, represents the action (integral of energy) because these elements are located on the diagonal with Σ = −2. This quantity is also preserved in the system in spite of the fact that the system is dissipative. Quod nota, the paradox is only illusory because two of the three elements of these specific circuits are, in principle, active elements, namely the *FDNR* and *FDNC* or the *linear FDNC* and *linear FDPC* elements.

The Hamiltonian of the circuit, containing only the HOEs from one diagonal, can be constructed via Equation (24). Equation (24) represents the known structure of a higher-order Hamiltonian, in which the generalized coordinates, generalized momenta, and higher-order Lagrangian appear. The Lagrangian is set up via the constitutive relations of the individual elements. The individual momenta are arranged according to (24) as sums of the generalized voltages of the corresponding orders across selected elements of the circuit. The Lagrangian is given as a sum of the state functions of the individual elements provided with the appropriate sign. The state function of the element corresponds to the area below its constitutive relation.

It has been newly found that the state function of the well-known HP memristor with the Joglekar window function for *p* = 1 is a composition of the dilogarithm and the exponential function.

The modeling diagram of the one-dimensional Σ-circuit from Figure 3 can be generalized in a classical way to a multi-dimensional system (e.g., a circuit with several loops), which can be used for implementation in the corresponding simulation program. For simulation, it is desirable to select a method for numerical integration that is suitable for the analysis of the sets of Hamilton’s differential equations [27]. The waveform of the Hamiltonian must exhibit negligible deflections from the constant value. This criterion provides a good feedback when seeking for an optimum configuration of the simulation task and parameters, including the time step.

The theoretical apparatus built in this work can also be used for circuits conisting only of two types of HOEs immediately neighboring each other on the common Σ-diagonal. However, the Hamiltonian of such a circuit is of a classical not a higher-order type. A typical example is a network of mutually interconnected memcapacitors and meminductors.

For researchers dealing with concrete memristive devices, for example the TiO_x_, TaO_x_, SiO_x_, HfO_x_ and other types, it will be useful to determine whether the presented Hamilton’s formalism for higher-order elements can also be applied to these devices.

It follows from the essence of the given formalism that this formalism can be used only for circuits that are made up of two-terminal HOEs, whereas all the HOEs from the circuit must be located on the common Σ-diagonal of Chua’s table. The question is whether the models of these memristive systems can be built from the above HOEs and whether all the other elements from the circuit are also located on the common diagonal.

Historically, the first model of the HP memristor with a simple window function [7,28] for modeling the nonlinear dopant drift can be classified as an ideal generic memristor. Since this memristor is equivalent to an ideal memristor, it can be considered an HOE of the (−1,−1) type, which complies with Hamilton’s formalism. However, the corresponding application circuit must contain, in addition to this memristor, only the other appropriate HOEs from the Σ = −2 diagonal—for example, (−2, 0), (0, −2) and other elements. If some of these elements are linear, then they can be moved in Chua’s table along the corresponding Δ-diagonals [31]. Then, the set of these admissible linear elements will grow, as is illustrated in Section 5.

However, the complex physical models of the above mentioned memristive devices, such as the Tunneling barrier model, TEAM, and others [32], are classified as extended memristors, which cannot be generally built from two-terminal HOEs. Thus, attempts to utilize the presented Hamilton’s formalism for such systems interfere with the principal limits. When looking for a Hamilton’s formalism suitable for these elements, it will be necessary to use other methods, such as those mentioned, for example, in [21,22].

## Figures and Tables

**Table 1:** The SPICE code for the numerical inversion of the constitutive relation $f_{2}(\ddot{x})$ in (44). For example, for *f*_2_ = 120.9 (see the last line of the code), SPICE computes the voltage at node xdd, V(xdd) = 4 mV, which is equal to the value of the charge $\ddot{x}$. For simplicity, definitions of the parameters of function *h* are omitted. The function *logexp* prevents exponential overflow in (49).

.func logexp(x,A) {if(abs(x)<50,ln(1+A*exp(x)),ln(A)+x)}
.func h(f2,xdd)={xdd/Roff+delta/(4*k*Roff)*(−ln(B)+logexp(4*k*f2,A))}
Exdd xdd 0 value={h(v(xdd),120.9)}
